# Identification and Characterization of Cell Lines HepG2, Hep3B217 and SNU387 as Models for Porcine Epidemic Diarrhea Coronavirus Infection

**DOI:** 10.3390/v14122754

**Published:** 2022-12-10

**Authors:** Lilei Lv, Huaye Luo, Lingxue Yu, Wu Tong, Yifeng Jiang, Guoxin Li, Guangzhi Tong, Yanhua Li, Changlong Liu

**Affiliations:** 1 Shanghai Veterinary Research Institute, Chinese Academy of Agricultural Sciences, Shanghai 200241, China; 2 Jiangsu Co-Innovation Center for the Prevention and Control of Important Animal Infectious Disease and Zoonosis, Yangzhou University, Yangzhou 225009, China; 3 College of Veterinary Medicine, Yangzhou University, Yangzhou 225009, China

**Keywords:** porcine epidemic diarrhea coronavirus, liver cell line, susceptibility, innate immune response

## Abstract

Porcine epidemic diarrhea virus (PEDV), a member of the genera alphacoronavirus, causes acute watery diarrhea and dehydration in suckling piglets and results in enormous economic losses in the swine industry worldwide. Identification and characterization of different cell lines are not only invaluable for PEDV entry and replication studies but also important for the development of various types of biological pharmaceuticals against PEDV. In this study, we present an approach to identify suitable permissive cell lines for PEDV research. Human cell lines were screened for a high correlation coefficient with the established PEDV infection model Huh7 based on RNA-seq data from the Cancer Cell Line Encyclopedia (CCLE). Experimentally testing permissiveness towards PEDV infection, three highly permissive human cell lines, HepG2, Hep3B217, and SNU387 were identified. The replication kinetics of PEDV in HepG2, Hep3B217, and SNU387 cells were similar to that in Vero and Huh7 cells. Additionally, the transcriptomes analysis showed robust induction of transcripts associated with the innate immune in response to PEDV infection in all three cell lines, including hundreds of inflammatory cytokine and interferon genes. Moreover, the expression of inflammatory cytokines and interferons were confirmed by qPCR assay. Our findings indicate that HepG2, Hep3B217, and SNU387 are suitable cell lines for PEDV replication and innate immune response studies.

## 1. Introduction

Porcine epidemic diarrhea virus (PEDV) is a highly infectious pathogen that can cause severe diseases in pigs with 80% to 100% mortality in infected piglets and results in enormous economic losses in the swine industry worldwide. PEDV was first described in the 1970s in England [1] and is a member of the α genus of the coronavirus family [2], which also includes porcine transmissible gastroenteritis coronavirus (TGEV), human NL63 coronavirus (HCoV-NL63), and bat coronavirus 512/2005 (BtCoV/512/2005).

PEDV is an enveloped, single-stranded, positive-sense RNA virus, which primarily infects and replicates in the villous enterocytes of the swine small intestine [3]. Its infection results in the destruction of the intestinal epithelium with subsequent villus shortening causing watery diarrhea that lasts for about a week [4]. Vero cells from African green monkey kidneys have been used for PEDV isolation and propagation. However, Vero cells are incapable of producing type I interferon in response to viral infections since a homozygous ∼9-Mb deletion on chromosome 12 causes the loss of the type I interferon gene cluster [5,6]. IPEC-J2 cells are also used for PEDV investigation, but the PEDV infectivity in IPEC-J2 cells is low because of the heterogeneity of these cells. A homogeneous cell line, designated IPI-FX, obtained from IPI-2I cells by sub-cloning with limited serial dilutions, was found to be highly susceptible to PEDV [7]. Other immortalized cell lines permissive for PEDV include MARC-145, LLC-PK1, PK-15, Huh7, ST, and so forth [8,9,10].

PEDV entry into host cells is mediated by the spike glycoprotein, which is considered as the main determinant of viral host and tissue tropism. It has been reported that PEDV infects multiple cell lines from different species including bat, rat, human, duck, and monkey in vitro. In addition to Huh7 cells, human embryonic kidney 293 (HEK293) cells are susceptible to infection with the PEDV vaccine strain CV777 and field isolate LNCT2 [11]. Chen et al. identified rat crypt epithelial cells (IEC-6) was highly susceptible to PEDV. PEDV could efficiently replicate in IEC-6 cells as it does in Vero cells [12]. Vero-cell-adapted PEDV was able to replicate in both primary alveolar macrophages and the continuous porcine alveolar macrophage line [13]. Interestingly, PEDV can infect and replicate in the immortalized duck intestinal epithelial cell (MK-DIEC) line, which was generated from the intestinal tissues of 19-day-old white Pekin duck embryo [14]. These findings implicate PEDV as a potential threat to other species and suggest antiviral strategies to control its spread.

Continuous cell lines that originate from mammalian tissues are for the production of various types of biological pharmaceuticals. Identification and characterization of different cell lines that are susceptible to PEDV are critical to understanding the entry of PEDV into cells and to facilitate the discovery of the functional receptors for PEDV, which are not determined yet [15]. In this study, we present an approach to identify suitable permissive cell lines for PEDV research. Aside from Huh7, three highly permissive human cell lines, HepG2, Hep3B217, and SNU387, were identified. Cell lines were characterized in more detail offering a broader choice of PEDV infection models in vitro.

## 2. Materials and Methods

### 2.1. CCLE RNA-Seq Dataset Analysis

The up-to-date processed CCLE (Cancer Cell Line Encyclopedia) expression data (CCLE_expression.csv) and cell lines annotation data (sample_info.csv) were downloaded from the CCLE database (https://depmap.org/portal/download/all/, accessed on 9 August 2021). The gene expression level was presented as log_2_ (TMP + 1) (TMP, transcripts per million) for each gene. Pearson’s correlation coefficient of each cell line with Huh7 transcriptome was calculated using the cor function with default parameters in R (v4.2.1) (https://www.r-project.org/).

### 2.2. Cells, Viruses, and Antibodies

Vero CCL-81 (ATCC), Huh7 (Procell, Wuhan, China), HepG2 (Procell), Hep3B217 (known as Hep 3B2.1-7, Procell), and BHK21 (Procell) were cultured in DMEM (Hyclone, Shanghai, China) containing 10% fetal bovine serum (FBS) (Gibco, Shanghai, China) and 1% penicillin/streptomycin (Gibco). Li-7 (Procell), SNU182 (MeiSenCTCC, Hangzhou, China), SNU387 (MeiSenCTCC), and SNU761 (MeiSenCTCC) were cultured in RPMI 1640 (Hyclone, Shanghai, China) supplemented with 10% FBS (Gibco) and 1% penicillin/streptomycin (Gibco). The PEDV-SD strain was isolated and stored in our laboratory. Recombinant rPEDV-EGFP was produced by transfecting infectious cDNA clones into BHK21 cells as previously described [16]. The viruses were titrated on the Vero CCL-81 cells by TCID_50_. Rabbit monoclonal antibody against PEDV N was purchased from Shanghai Ango Biotech.

### 2.3. TCID_50_ Assay

Vero CCL-81 cells were seeded into 96-well plates and cultured to reach 90% confluence. The cell monolayers were washed twice with maintaining medium containing 5 μg/mL trypsin (Thermo Fisher Scientific, Shanghai, China). One hundred microliters of ten-fold dilutions of virus samples were inoculated in eight replicates per dilution. Viral CPE was monitored for 3–5 days, and virus titers were calculated using the Reed–Muench method and expressed as TCID_50_ per milliliter.

### 2.4. RNA Extraction and RT-qPCR Assay

Total RNA was purified from different cell lines infected with PEDV-SD or mock using TRIzol (Thermo Fisher Scientific, Shanghai, China). The reverse transcription reaction was carried out with 1 μg of total RNA using a PrimeScript first-strand cDNA synthesis kit (Takara, Beijing, China). Quantitative real-time PCR was performed using SYBR Premix Ex Taq (Takara, Beijing, China) and a LightCycler 96 instrument (Roche, Shanghai, China). The relative quantities of the genes were calculated using GAPDH as a reference, using the formula: 2^−[Ct(Gene)−Ct(GAPDH)]^. The primer sequences for each gene are provided in Table 1.

### 2.5. Infection of Different Cell Lines with rPEDV-EGFP

Huh7, HepG2, Hep3B217, SNU387, SNU761, SNU182, and Li-7 cells were precultured in 12-well plates to 80% confluence and infected with rPEDV-EGFP at an MOI of 0.1. After 2 h of incubation, the supernatant containing virus was discarded. Cells were washed three times with PBS and cultured in fresh DMEM supplemented with 2% FBS. EGFP expression was observed at 48 h after rPEDV-EGFP infection by fluorescence microscope (Zeiss, Germany).

### 2.6. Viral Growth Curve

The different type cells were cultured in 6-well plates and infected with PEDV-SD at a multiplicity of infection (MOI) of 0.1. Cells were incubated with 10-fold diluted virus supernatant with DMEM for 2 h, then washed twice with PBS, and supplemented with 1 mL DMEM with or without 5 μg/mL of trypsin. Virus supernatants of two wells for each virus were collected at 12, 24, 36, 48, 60, and 72 hpi for virus titration by TCID_50_. The viral growth curves were generated with GraphPad Prism 9.

### 2.7. Viral Plaque Assay

Different cell lines including Vero, Huh7, HepG2, Hep3B217, and SNU387 were precultured in 6-well plates to 100% confluence and incubated with 10-fold dilutions of PEDV for 2 h. The cells were washed three times with PBS and cultured in DMEM supplied with 1% of ultrapure low melting point agarose (Thermo Fisher Scientific, Shanghai, China), and then inverted after the medium was completely solidified. Three days post-infection, cells were fixed with 4% paraformaldehyde and stained with 1% crystal violet to visualize plaques.

### 2.8. Indirect Immunofluorescence Assay

Cells were seeded in 6-well plates. When they reached 80% confluency, cells were infected with PEDV-SD at an MOI of 0.01. After 24 h post-infection, cells were washed with cold PBS three times before being fixed with cold methanol for 10 min, and then the cells were incubated with 5% BSA at 37 °C for 1 h prior to primary antibody (Rabbit PEDV N monoclonal antibody, 1:800) incubation for 1.5 h, followed by incubating with donkey anti-rabbit IgG (H+L) Alexa Fluor 594 (Thermo Fisher Scientific; 1:1000) for 1 h. Cell nuclei were stained with DAPI (cat#: D9542, Sigma) for 5 min at room temperature. The images were captured with a fluorescence microscope (Zeiss, Germany).

### 2.9. RNA-Seq and Analysis

The three cell lines, HepG2, Hep3B217, and SNU387, as well as Huh7 were infected with PEDV at an MOI of 0.01. PEDV- and mock-infected samples were collected with two biological replicates for all four cell lines. Total RNA was extracted from porcine intestinal organoids using the miRNeasy Minikit (Qiagen, Shanghai, China) at 6 h post-infection. Total RNAs were sent to the OEbiotech (Shanghai, China) for sequencing including library construction. We obtained 40 million at least of 150 bp paired-end reads for each RNA sample. Clean reads were obtained from raw reads by filtering out poor quality reads and removing adapters contamination using Cutadap (v1.16), and reads with <10% quality threshold and <20 Phred score were also removed. Then, clean reads were mapped to the human reference genome (GRCh38/hg38) using HISAT2 [17]. Read count tables were generated using HTSeq (v0.6.1) [18]. Differentially expressed genes (DEGs) between PEDV infection and mock were evaluated using DESeq2 (v1.36.0) [19] at a significance cutoff of FDR (false discovery rate) *p* < 0.01 unless otherwise stated. All of the downstream statistical analyses and generating plots were performed in R (v4.2.1). Pearson’s coefficient was calculated using the cor function with default parameters in R. Heatmaps were generated using ComplexHeatmap [20] based on the z-score of FPKM values. Gene over representation analysis and gene set enrichment analysis (GSEA) [21] were performed with the clusterProfiler package [22] based on the KEGG pathway. KEGG pathways with adjusted *p*-values < 0.05 were considered to be significantly enriched. All enrichment plots were generated using enrichplot package. Venn diagram was generated using VennDiagram package.

### 2.10. Statistical Analysis

All data were analyzed with R and GraphPad Prism 9 (GraphPad, San Diego, CA, USA) and provided as mean ± SD unless otherwise indicated. Statistical analyses were performed using an unpaired Student’s *t*-test. The significance level (*p* value) was set at <0.05 (*), <0.01 (**), and <0.001 (***).

## 3. Results

### 3.1. Selection of Cell Lines for PEDV Infection

**Figure 1:**
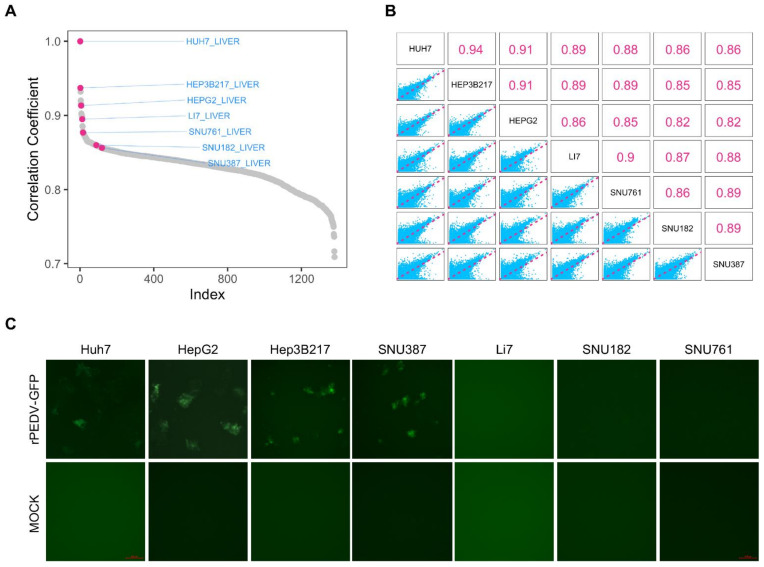
Selection of cell lines for PEDV infection. (**A**) Cell lines rank ordered by Pearson’s correlation coefficient with Huh7 from CCLE database. Red dots: cell lines were chosen in this study. Gray dots: other cell lines. (**B**) Correlation plot of seven cell lines. Upper panel: pairwise correlation coefficients of seven cell lines. Lower panel: pairwise dot plots of seven cell lines based on cell transcriptome. (**C**) Representative images for GFP expression 48 h after rPEDV-GFP infection of different cell lines.

The physiological state of a cell is an approximate reflection of its transcriptome, it is a reasonable assumption that similar global gene expression can be highly correlated with cell physiological state. The Cancer Cell Line Encyclopedia (CCLE) provides large-scale mRNA-seq expression data for over 1300 cell lines. Since Huh7 is an established cell culture infection model for PEDV [8,23], we first screened for transcriptomes close to Huh7 in the CCLE dataset based on Pearson’s correlation coefficient, which enabled us to identify potential susceptible cell lines prone to PEDV entry. We chose six liver carcinoma cell lines including HepG2, Hep3B217, Li7, SNU182, SNU761, and SNU387, which are human liver epithelial cells. Among these cell lines, the transcriptomes of HepG2 and Hep3B217 were highly correlated with that of Huh7 with the correlation coefficients of 0.91 and 0.90, respectively, whereas the correlation coefficients of Li7, SNU182, SNU387, and SNU761 with Huh7 were lower than 0.85 (Figure 1A). Additionally, the correlation coefficients among these six cell lines as well as Huh7 were calculated based on their transcriptomes (Figure 1B). Since the liver carcinoma cell line Huh7 is shown to be permissive for PEDV [8,24], it was used as a positive control cell line. Then, the cell lines were exposed to a reporter virus (rPEDV-EGFP) engineered to express enhanced GFP (EGFP) during replication. This reporter virus replicated well in Vero CCL-81 cells [16]. We repeatedly detected some EGFP expression in HepG2, Hep3B217, and SNU387 cells as well as the Huh7 cells, whereas there was no GFP expressed in Li7, SNU182, and SNU761 cells (Figure 1C). These results suggest that HepG2, Hep3B217, and SNU387 support PEDV replication.

### 3.2. PEDV Can Infect HepG2, Hep3B217, and SNU387 Cells

**Figure 2:**
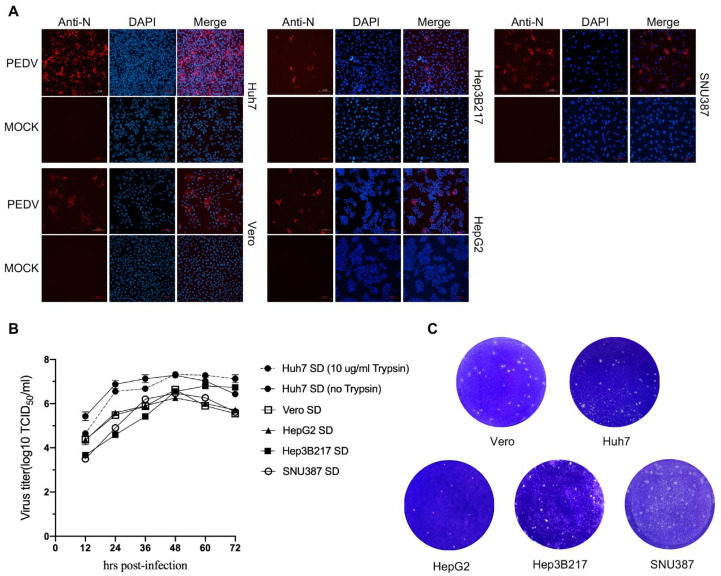
PEDV infections in different cell lines cell. (**A**) PEDV-SD was used to infect different cell lines at an MOI of 1.0. Cells were fixed with 4.0% paraformaldehyde and 0.2% glutaraldehyde 24 h post-infection. PEDV was detected by Alexa Fluor 594 labeled anti-PEDV N protein antibody and observed under a fluorescence microscope. Representative images are shown (**B**) The growth curves of PEDV-SD strain in Huh7, Vero, HepG2, Hep3B217, and SNU387 cells. (**C**) The plaque assay of PEDV-SD strain in Huh7, Vero, HepG2, Hep3B217, and SNU387 cells. Representative images are shown.

To confirm that this above result was not unique to the GFP-expressing PEDV, HepG2, Hep3B217, and SNU387 cells were infected with the field strain PEDV-SD isolated by our group [25], which is a trypsin-independent PEDV strain (Figure 2B), at an MOI of 0.01 and observed for cytopathic effect (CPE) under a microscope. Typical CPE was observed in all three cell lines at 24 h post-infection. The PEDV-infected cells were round and detached. However, the mock-infected cells remained with normal morphology during PEDV infection. Then, the infection was further confirmed by an immunofluorescence assay using a monoclonal antibody against PEDV nucleocapsid (N) protein. The results showed that strong PEDV-N-positive signals were observed in HepG2, Hep3B217, and SNU387 cells infected with the PEDV-SD strain (Figure 2A) indicating these cell lines were successfully infected by PEDV.

To test and compare the replicative capacity of PEDV in different cell lines, the PEDV replication kinetics were measured in these three cell lines as well as Huh7 and Vero cells. Cells were seeded in the 6-well plate and inoculated with strain PEDV-SD at an MOI of 0.01. The supernatant was collected every 12 h and used for viral titration. The PEDV-SD strain exhibited a robust and similar growth curve in Huh7, HepG2, Hep3B217, and SNU387 as well as Vero cells, showing a higher viral titer ranging from 10^6^ TCID_50_/_mL_ to 10^7^ TCID_50_/_mL_ (Figure 2B). We also tested the ability of PEDV to form plaques in these cell lines by plaque assay. The data showed that the infection of the PEDV-SD strain formed plaques in all cell lines. The plaque sizes from Vero, SNU387, and Hep3B217 were similar and larger than that from Huh7 and HepG2, which were small (Figure 2C). Taken together, the above data showed that HepG2, Hep3B217, and SNU387 cells supported PEDV infection and subsequent replication.

### 3.3. Transcriptome Analysis of HepG2, Hep3B217, and SNU387 Infected with PEDV

**Figure 3:**
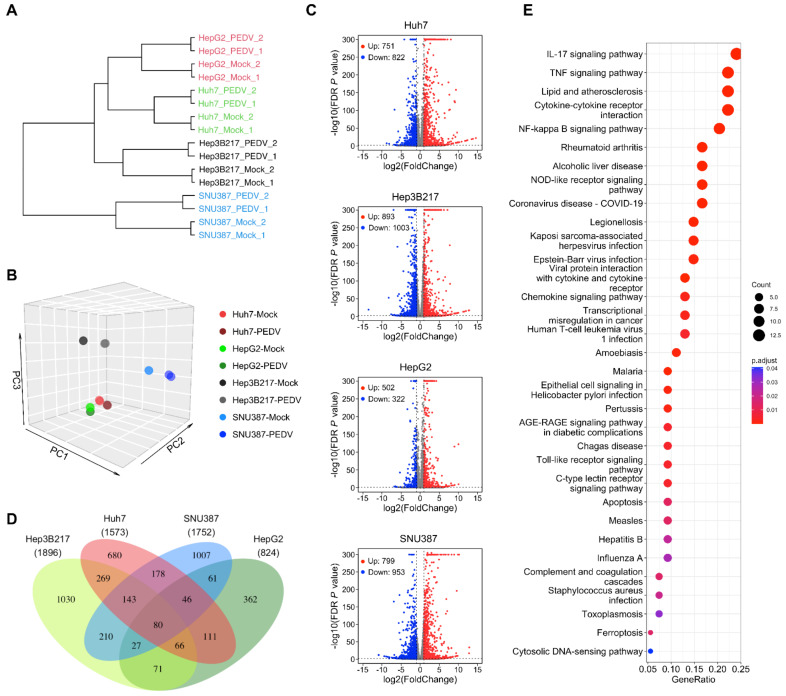
Transcriptional response to PEDV infection in different cell lines. (**A**) The dendrogram shows the hierarchal clustering of global gene expression between the different cell lines in regards to time post-infection for PEDV infection. The Pearson correlation was used to generate the distance matrix. (**B**) PCA analysis was performed on the FPKM expression matrix of all samples. (**C**) The transcriptomes of different cell lines infected with PEDV were compared with mock-infected cultures at 6 hpi. Volcano plots depict the differential gene expression for each cell line (red and blue as linear fold change of 2 and FDR *p* < 0.01). (**D**) Venn diagram showing overlapped DEGs in PEDV- infected Huh7, HepG2, Hep3B217, and SNU387 compared to their corresponding mock-infected control. (**E**) KEGG pathway enrichment analysis of overlapped DEGs in all PEDV-infected cell lines. The vertical axis shows the functional classification, and the horizontal axis shows the GeneRatio.

With the goal of better understanding how HepG2, Hep3B217, and SNU387 respond to PEDV infection at an early stage, we performed RNA sequencing (RNA-seq) experiments using HepG2, Hep3B217, and SNU387 as well as the Huh7 control infected with 0.01 MOI of PEDV and evaluated the transcript landscape of these cell lines at 6 h after viral challenges. PEDV- and mock-infected samples were collected with two biological replicates for all four cell lines. The correlation of PEDV- and mock-infected cell lines were calculated based on all genes expression (FPKM). As shown in Figure 3A, the same cell lines were clustered together. Consistent with the sample correlation analysis, we observed that RNA-seq samples from different cell lines could be distinguished when visualized by principal component analysis (PCA) (Figure 3B). We first examined changes in gene expression induced upon PEDV infection compared to mock infection. With the threshold of false discovery rate (FDR) corrected *p* value < 0.01 and |log2 (fold change)| > = 1, the numbers of differentially expressed genes (DEGs) in PEDV-infected cell lines were identified compared to mock-infected cell lines. The volcano plots of the results showed the significantly differentially expressed genes in different cell lines after PEDV infection. There were 1,573 DEGs (751 upregulated and 822 downregulated), 824 DEGs (502 upregulated and 322 downregulated), 1,896 DEGs (893 upregulated and 1003 downregulated), and 1,752 DEGs (799 upregulated and 953 downregulated) in PEDV-infected Huh7, HepG2, Hep3B217, and SNU387 compared to their corresponding mock-infected cells, respectively (Figure 3C and Supplemental Tables S1–S4). We then analyzed the overlapped DEGs from all four cell lines infected with PEDV versus mock and found DEGs in these cell lines were very different, having only 80 genes in common (Figure 3D). KEGG pathway enrichment analysis for the overlapped DEGs revealed that the most significantly regulated pathway in all PEDV-infected cell lines was the IL-17 signaling pathway. In addition, the TNFα, NF-kappa B, cytokine–cytokine-receptor interaction, NOD-like receptor, and viral protein interaction with cytokine and cytokine receptor signaling pathways are among the top upregulated pathways (Figure 3E).

**Figure 4:**
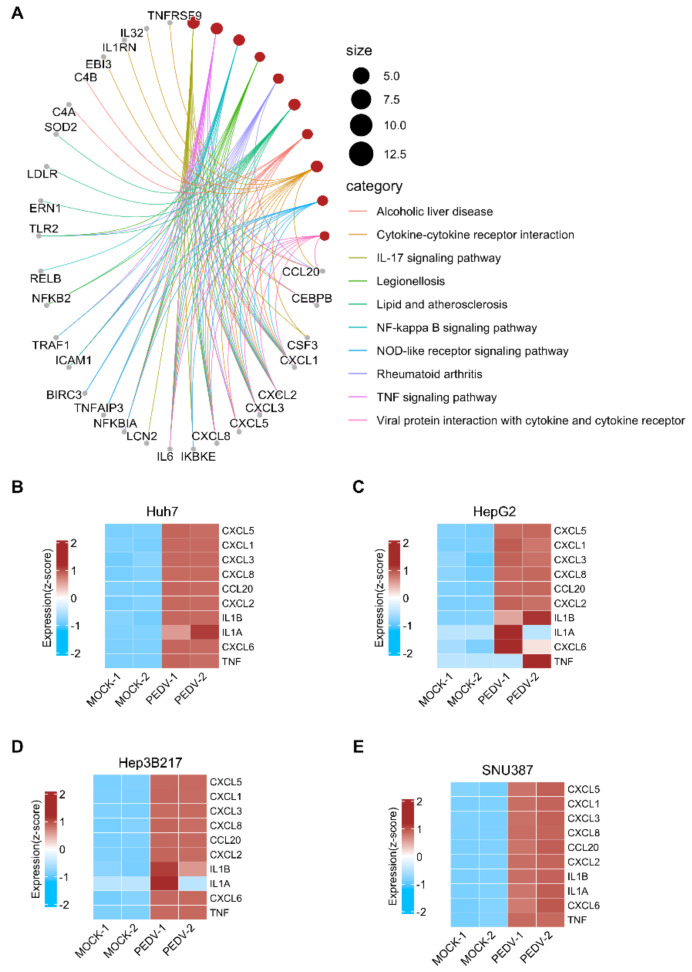
Cytokine and chemokine gene expression after PEDV infection in different cell lines. (**A**) The network plot shows that the linkages of genes and pathways for the top 10 pathways from KEGG pathway enrichment analysis of common DEGs in all cell lines compared to their control. Heatmaps depicting virally regulated cytokines reported in [26,27] upon PEDV infection in Huh7 (**B**), HepG2 (**C**), Hep3B217 (**D**), and SNU387 (**E**) cell lines. Colored bar represents z-score of log2 transformed values.

PEDV infection induces inflammatory cytokines and chemokine in Vero cells [26]. We further checked the inflammatory cytokines and chemokine genes that are involved in the cellular response to the top 10 pathways in the KEGG pathway enrichment analysis (Figure 4A) and found that cytokine or chemokine genes including CXCL1, CXCL2, CXCL3, CXCL5, CXCL8 (IL8), CCL20, IL6, and CXCL6, which were induced by PEDV in Vero [26] or by enterovirus in human intestinal organoids [27], were upregulated in all PEDV-infected cell lines. See Figure 4B–E, heatmaps depict virally regulated cytokines reported in the previous report [26,27] upon PEDV infection in all four cell lines from RNA-seq data.

### 3.4. PEDV Infection Induces Host Innate Immune Responses in HepG2, Hep3B217, and SNU387 Cells

**Figure 5:**
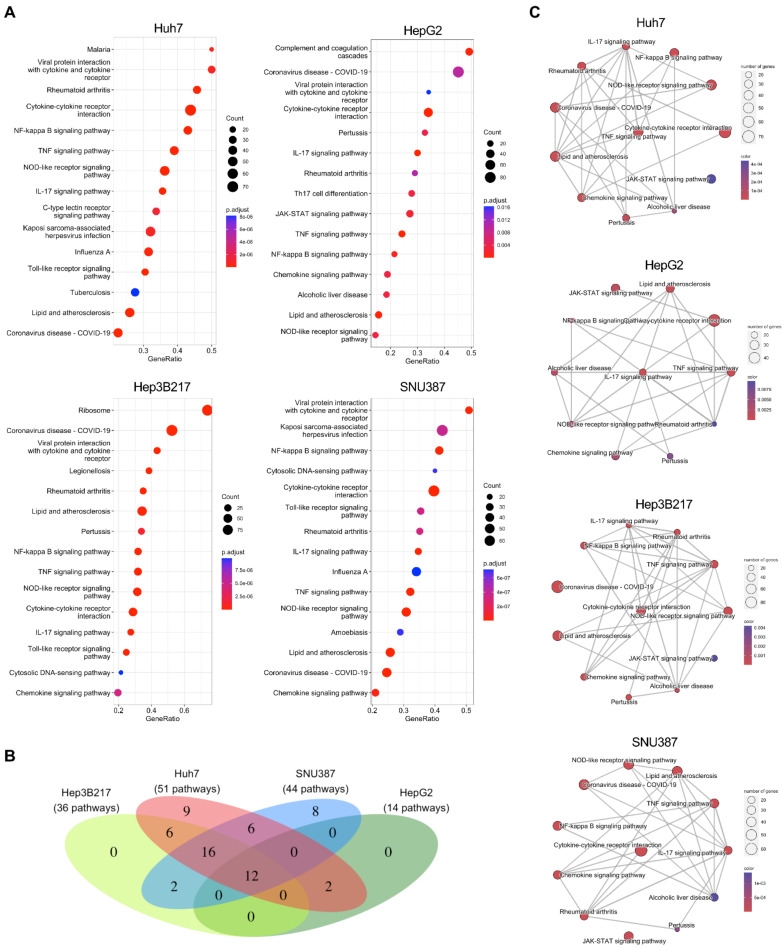
PEDV infection induces transcripts associated with innate immune responses in different cell lines. (**A**) GSEA analysis of all genes expressed in different cell lines infected with PEDV versus mock based on KEGG pathways. The vertical axis shows the functional classification, and the horizontal axis shows the GeneRatio. (**B**) Venn diagram showing overlapping enriched KEGG pathways in GSEA analysis from PEDV-infected Huh7, HepG2, Hep3B217, and SNU387 compared to their mock-infected control. (**C**) Enrichment map showing the network of 12 commonly enriched KEGG pathways with edges connecting overlapped gene sets.

To investigate the molecular landscape of the host innate immune response to PEDV infection in all three newly identified cell lines, we then performed gene sets enrichment analysis (GSEA) to evaluate the global change of transcriptome for all three cell lines as well as for the positive control Huh7 in response to PEDV infection. GSEA analysis based on the KEGG database showed that there were 51, 14, 36, and 44 pathways with significant enrichment (adjust *p* < 0.01) in PEDV-infected-Huh7, -HepG2, -Hep3B217, and -SNU387 compared to their corresponding control, respectively. The top 15 significant enrichment pathways were shown (Figure 5A. Tables S5–S8). We overlapped all these enrichment pathways from different cell lines and there were 12 pathways which overlapped (Figure 5B). Most of these overlapped pathways were inflammatory and innate immune related including the cytokine–cytokine-receptor interaction pathway, NOD-like receptor signaling pathway, TNF signaling pathway, IL-17 signaling pathway, NF-kappa B signaling pathway, chemokine signaling pathway, and JAK–STAT signaling pathway (Figure 5C). The coronavirus disease (COVID-19) pathway also ranked top of the significant enrichment pathways in all PEDV-infected cell lines.

**Figure 6:**
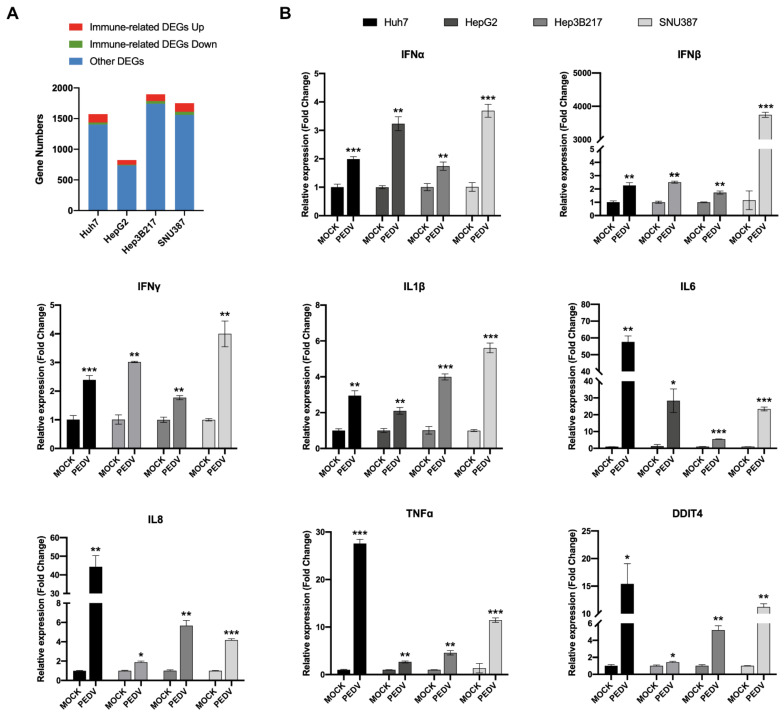
The host immune response to PEDV-SD infection in different cell lines. (**A**) The stacked bar plot shows the immune-related DEGs numbers in PEDV-infected cell lines. (**B**) The RT-qPCR analysis of IFNα, IFNβ, IFNγ, IL1β, IL6, IL8 (CXCL8), TNFα, and DDIT4 in different cell lines infected with the PEDV-SD strain. Error bars represent standard deviations of technical triplicates. * *p* < 0.05, ** *p* < 0.01, *** *p* < 0.001, n = 3 per group.

The first line to combat viral infections is the host innate immune system, which is related to multiple proteins and pathways including inflammatory cytokines, interferons, and so on. To explore the alteration of innate-immune-related genes in host cells after PEDV infection in these cell lines at an early stage, the innate-immune-related genes in the DEGs were further screened based on the innate immune genes list of the InnateDB (http://www.innatedb.ca/redirect.do?go=aboutIDB, accessed on 13 August 2022) which provides a total of 1,040 curated human genes related to the innate immune response. Among the DEGs in PEDV-infected cell lines, there were 171, 89, 153, and 192 immune-related genes in PEDV-infected Huh7, HepG2, Hep3B217, and SNU387 compared to their mock controls, respectively. Among all innate-immune-related DEGs, most of them were upregulated (Figure 6A). To validate the accuracy of immune-related gene expression levels in the RNA-seq results, qRT-PCR experiments were performed for eight genes including IFNα1, IFNβ1, IFNγ, IL1β, IL6, TNFα, CXCL8 (IL8), and DDIT4, which are known to play critical roles in the innate immune response. As shown in Figure 6B, expression levels of the eight genes exhibited high consistencies with those of RNA-seq, which supported that the gene expression quantification from RNA-seq was reliable. Collectively, these results demonstrated that all three newly identified cell lines were able to generate a robust immune response against PEDV infection suggesting that these three cell lines are suitable models for the in vitro study of the innate immune response to PEDV infection.

## 4. Discussion

Cell lines provide invaluable information on viral pathogenesis and its interplay with the innate immune response. The lack of suitable cell lines is therefore one of the major impediments to progress in the study of PEDV. In the current study, we have systematically identified and characterized PEDV-permissive human liver cell lines that have never been published before. Three of the six human cell lines tested were highly permissive for PEDV (Figure 1 and Figure 2). Unlike the immortalized cell line models, organoids are self-organizing 3D structures grown from adult stem cells and recapitulate key aspects of the organ from which those cells derive [28]. Our group and others have developed several culture systems for porcine intestinal organoids for studying porcine enteric coronaviruses in vitro [25,29,30]. Porcine intestinal organoids recapitulate the events of PEDV infection that occur in vivo and provide a valuable in vitro model for studying PEDV infection. However, intestinal organoids may not allow the production of high titer of PEDV in vitro. HepG2, Hep3B217, and SNU387 cell lines could support PEDV replication with a similar titer to that in Vero cells (Figure 2B). These three cell lines may substitute Vero cells for virus preparations for vaccine preparation of PEDV. However, the antigenicity of PEDV from these cell lines needs to be further investigated.

Viral permissiveness includes the ability of the virus to enter the cell, replicate, and release infectious progeny. The lacking permissiveness for PEDV of these three cell lines, Li7, SNU182, and SNU761, is likely to be explained by the following reasons. First, the expression of cellular receptors of PEDV entry may be absent from these non-permissive cell lines or genetic variation inactivated the PEDV entry factor. Second, several numbers of cellular processes can effectively interfere with the PEDV replication cycle including the innate immune response. The underlying mechanism of the lacking permissiveness for PEDV of these cell lines needs to be further investigated. It is worth noting that of the three newly identified PEDV-susceptible cells, the Hep3B217 cell line contains an integrated hepatitis B virus genome. Besides direct expression alteration of HBV genome integration in Hep3B217, viral insertion in the Hep3B217 genome can also lead to genomic instability and introduce copy number changes. This may explain why Hep3B217 had the most DEGs after PEDV infection [31].

PEDV primarily infects and replicates in the villous enterocytes of the swine small intestine. Meanwhile, several cell types from different species including bat, rat, human, duck, and monkey have been reported to be infected by PEDV. These findings suggest that the virus utilizes the evolutionarily conserved cell components as receptors, implicating PEDV as a potential threat to other species. The PEDV S1 subdomain has been shown to bind to sialic acid glycans [8,32]. Porcine aminopeptidase N (pAPN) was suggested as a functional receptor for PEDV [8,33]. However, the use of pAPN as a receptor for PEDV has been questioned [15,23,34,35]. Therefore, the cell lines identified in this study could facilitate the discovery of a functional receptor of PEDV by analyzing the different gene expression between the transcriptome of PEDV-permissive and -nonpermissive cell lines such as Li7, SNU182, and SNU761.

Vero cells from African green monkey kidneys have been commonly used for PEDV isolation and propagation. However, Vero cells are incapable of producing type I interferon in response to viral infections. Therefore, Vero is not the ideal cell type to study the molecular mechanisms of PEDV infection and the viral modulation of innate immune responses. Unlike the Vero cells, our RNA sequencing analysis demonstrated that PEDV infection induced cytokines and interferons in HepG2, Hep3B217, and SNU387 cell lines. Tumor necrosis factor alpha (TNFα) is a well-recognized proinflammatory cytokine that is critical in the innate host defense against infectious agents. There were increased pro-inflammatory cytokine (TNFα, IL-6, IL-8, IL-12, and IL-17) [36,37] responses in acutely PEDV-infected neonatal piglets. This coincided with increased numbers of PEDV-antigen-positive cells [38] or inflammatory cells (lymphocytes or neutrophils) in the gut-associated lymphoid tissue (GALT) [39]. In line with the previous report, PEDV infection of HepG2, Hep3B217, and SNU387 activated several inflammatory cytokines including TNFα, IL-6, IL-8, and IL-17. Our RNA-seq analysis also showed that PEDV-infected HepG2, Hep3B217, and SNU387 could induce several cytokines that were involved in inflammatory cells’ expansion and migration implying that those cell lines were suitable in vitro models for studying the innate immune response of PEDV infection. It has been reported that the expression levels of chemokines including IL-8, CXCL9, CXCL10, and CXCL13 are significantly increased in the ileum of PEDV-infected piglets [40]. Our RNA-seq analysis found that the most upregulated chemokine genes in all these cell lines were CXCL1, CXCL2, CXCL3, CXCL5, CXCL8 (IL8), CCL20, IL6, and CXCL6, and most of these chemokines were not reported before. These chemokines may be specifically induced in PEDV-infected liver epithelial cells. Previous transcriptomic analysis in PEDV-infected IPEC-J2 cells suggests that STAT transcription factors may serve as key regulators in the response to PEDV infection [41]. Our KEGG pathway enrichment analysis also showed that the JAK–STAT signaling pathway was significantly induced in all three new identified cell lines (Figure 3 and Figure 4). Likewise, responses to interferon-alpha and -gamma were activated in PEDV-infected cell lines.

In conclusion, our studies identified HepG2, Hep3B217, and SNU387 cells were highly susceptible to PEDV. PEDV could efficiently replicate in these cell lines as it does in Vero cells. Moreover, PEDV infection activated potent inflammatory cytokines and interferons in HepG2, Hep3B217, and SNU387 cells, which highly simulated its infection in the small intestine and in Vero cells. All these data above suggest that HepG2, Hep3B217, and SNU387 cell lines can be used as suitable models for PEDV studies.

## Figures and Tables

**Table 1:** Primers used in this study.

Primer Name.	Sequence (5′-3′)	Reference No.
IL8-F	TAGCAAAATTGAGGCCAAGG	NM_000584.4
IL8-R	GGACTTGTGGATCCTGGCTA	
DDIT4-F	TGTTTAGCTCCGCCAACTCT	NM_019058.4
DDIT4-R	CACCCCAAAAGTTCAGTCGT	
IFNα-F	CTGGGAGGTTGTCAGAGCAG	NM_024013.3
ΙFΝα-R	ATGAAAGCGTGACCTGGTGT	
IFNβ-F	TGCTCTGGCACAACAGGTAG	NM_002176.4
IFNβ-R	AGCCTCCCATTCAATTGCCA	
IFNγ-F	GGCTTTATCTCAGGGGCCAA	NM_000619.3
IFNγ-R	GCACCAGGCATGAAATCTCC	
IL1β-F	ACGATGCACCTGTACGATCA	NM_000576.3
IL1β-R	TCTTTCAACACGCAGGACAG	
IL6-F	TTCGGTCCAGTTGCCTTCTC	NM_000600.5
IL6-R	TGTTTTCTGCCAGTGCCTCT	
TNFα-F	CACAGTGAAGTGCTGGCAAC	NM_000594.4
TNFα-R	AGGAAGGCCTAAGGTCCACT	
GAPDH-F	GATTTGGTCGTATTGGGCGC	NM_002046.7
GAPDH-R	TTCCCGTTCTCAGCCTTGAC	

## Data Availability

The data that support the findings of this study are available from the corresponding author upon reasonable request.

## Supplementary Materials

The following are available online at https://www.mdpi.com/article/10.3390/v14122754/s1, Table S1: The differentially expressed genes between PEDV- and mock-infected Huh7, Table S2: The differentially expressed genes between PEDV- and mock-infected HepG2, Table S3: The differentially expressed genes between PEDV- and mock-infected Hep3B217, Table S4: The differentially expressed genes between PEDV- and mock-infected SNU387, Table S5: The significant enrichment KEGG pathways from GSEA analysis of PEDV- vs mock-infected Huh7, Table S6: The significant enrichment KEGG pathways from GSEA analysis of PEDV- vs mock-infected HepG2, Table S7: The significant enrichment KEGG pathways from GSEA analysis of PEDV- vs mock-infected Hep3B217, Table S8: The significant enrichment KEGG pathways from GSEA analysis of PEDV- vs mock-infected SNU387.

## References

[1] Wood E.N. (1977). An apparently new syndrome of porcine epidemic diarrhoea. Vet. Rec..

[2] Pensaert M.B., de Bouck P. (1978). A new coronavirus-like particle associated with diarrhea in swine. Arch. Virol..

[3] Ducatelle R., Coussement W., Pensaert M.B., Debouck P., Hoorens J. (1981). In vivo morphogenesis of a new porcine enteric coronavirus, CV 777. Arch. Virol..

[4] Lee C. (2015). Porcine epidemic diarrhea virus: An emerging and re-emerging epizootic swine virus. Virol. J..

[5] Desmyter J., Melnick J.L., Rawls W.E. (1968). Defectiveness of interferon production and of rubella virus interference in a line of African green monkey kidney cells (Vero). J. Virol..

[6] Osada N., Kohara A., Yamaji T., Hirayama N., Kasai F., Sekizuka T., Kuroda M., Hanada K. (2014). The genome landscape of the african green monkey kidney-derived vero cell line. DNA Res..

[7] Wang X., Fang L., Liu S., Ke W., Wang D., Peng G., Xiao S. (2019). Susceptibility of porcine IPI-2I intestinal epithelial cells to infection with swine enteric coronaviruses. Vet. Microbiol..

[8] Liu C., Tang J., Ma Y., Liang X., Yang Y., Peng G., Qi Q., Jiang S., Li J., Du L. (2015). Receptor usage and cell entry of porcine epidemic diarrhea coronavirus. J. Virol..

[9] Zhang Q., Shi K., Yoo D. (2016). Suppression of type I interferon production by porcine epidemic diarrhea virus and degradation of CREB-binding protein by nsp1. Virology.

[10] Zhang Q., Ma J., Yoo D. (2017). Inhibition of NF-kappaB activity by the porcine epidemic diarrhea virus nonstructural protein 1 for innate immune evasion. Virology.

[11] Zhang J., Guo L., Xu Y., Yang L., Shi H., Feng L., Wang Y. (2017). Characterization of porcine epidemic diarrhea virus infectivity in human embryonic kidney cells. Arch. Virol..

[12] Chen J., Cui Y., Wang Z., Liu G. (2020). Identification and characterization of PEDV infection in rat crypt epithelial cells. Vet. Microbiol..

[13] Park J.E., Shin H.J. (2014). Porcine epidemic diarrhea virus infects and replicates in porcine alveolar macrophages. Virus Res..

[14] Khatri M. (2015). Porcine epidemic diarrhea virus replication in duck intestinal cell line. Emerg. Infect. Dis..

[15] Li W., Luo R., He Q., van Kuppeveld F.J.M., Rottier P.J.M., Bosch B.J. (2017). Aminopeptidase N is not required for porcine epidemic diarrhea virus cell entry. Virus Res..

[16] Zhou Y., Li C., Ren C., Hu J., Song C., Wang X., Li Y. (2022). One-Step Assembly of a Porcine Epidemic Diarrhea Virus Infectious cDNA Clone by Homologous Recombination in Yeast: Rapid Manipulation of Viral Genome With CRISPR/Cas9 Gene-Editing Technology. Front. Microbiol..

[17] Kim D., Langmead B., Salzberg S.L. (2015). HISAT: A fast spliced aligner with low memory requirements. Nat. Methods.

[18] Anders S., Pyl P.T., Huber W. (2015). HTSeq—A Python framework to work with high-throughput sequencing data. Bioinformatics.

[19] Love M.I., Huber W., Anders S. (2014). Moderated estimation of fold change and dispersion for RNA-seq data with DESeq2. Genome. Biol..

[20] Gu Z., Eils R., Schlesner M. (2016). Complex heatmaps reveal patterns and correlations in multidimensional genomic data. Bioinformatics.

[21] Subramanian A., Tamayo P., Mootha V.K., Mukherjee S., Ebert B.L., Gillette M.A., Paulovich A., Pomeroy S.L., Golub T.R., Lander E.S. (2005). Gene set enrichment analysis: A knowledge-based approach for interpreting genome-wide expression profiles. Proc. Natl. Acad. Sci. USA.

[22] Wu T., Hu E., Xu S., Chen M., Guo P., Dai Z., Feng T., Zhou L., Tang W., Zhan L. (2021). clusterProfiler 4.0: A universal enrichment tool for interpreting omics data. Innovation.

[23] Ji C.M., Wang B., Zhou J., Huang Y.W. (2018). Aminopeptidase-N-independent entry of porcine epidemic diarrhea virus into Vero or porcine small intestine epithelial cells. Virology.

[24] Hofmann M., Wyler R. (1988). Propagation of the virus of porcine epidemic diarrhea in cell culture. J. Clin. Microbiol..

[25] Zhang M., Lv L., Cai H., Li Y., Gao F., Yu L., Jiang Y., Tong W., Li L., Li G. (2022). Long-Term Expansion of Porcine Intestinal Organoids Serves as an in vitro Model for Swine Enteric Coronavirus Infection. Front. Microbiol..

[26] Yu L., Dong J., Wang Y., Zhang P., Liu Y., Zhang L., Liang P., Wang L., Song C. (2019). Porcine epidemic diarrhea virus nsp4 induces pro-inflammatory cytokine and chemokine expression inhibiting viral replication in vitro. Arch. Virol..

[27] Drummond C.G., Bolock A.M., Ma C., Luke C.J., Good M., Coyne C.B. (2017). Enteroviruses infect human enteroids and induce antiviral signaling in a cell lineage-specific manner. Proc. Natl. Acad. Sci. USA.

[28] Clevers H. (2016). Modeling Development and Disease with Organoids. Cell.

[29] Li Y., Yang N., Chen J., Huang X., Zhang N., Yang S., Liu G., Liu G. (2020). Next-Generation Porcine Intestinal Organoids: An Apical-Out Organoid Model for Swine Enteric Virus Infection and Immune Response Investigations. J. Virol..

[30] Li L., Fu F., Guo S., Wang H., He X., Xue M., Yin L., Feng L., Liu P. (2019). Porcine Intestinal Enteroids: A New Model for Studying Enteric Coronavirus Porcine Epidemic Diarrhea Virus Infection and the Host Innate Response. J. Virol..

[31] Jiang Z., Jhunjhunwala S., Liu J., Haverty P.M., Kennemer M.I., Guan Y., Lee W., Carnevali P., Stinson J., Johnson S. (2012). The effects of hepatitis B virus integration into the genomes of hepatocellular carcinoma patients. Genome. Res..

[32] Li W., van Kuppeveld F.J.M., He Q., Rottier P.J.M., Bosch B.J. (2016). Cellular entry of the porcine epidemic diarrhea virus. Virus Res..

[33] Li B.X., Ge J.W., Li Y.J. (2007). Porcine aminopeptidase N is a functional receptor for the PEDV coronavirus. Virology.

[34] Shirato K., Maejima M., Islam M.T., Miyazaki A., Kawase M., Matsuyama S., Taguchi F. (2016). Porcine aminopeptidase N is not a cellular receptor of porcine epidemic diarrhea virus, but promotes its infectivity via aminopeptidase activity. J. Gen. Virol..

[35] Whitworth K.M., Rowland R.R.R., Petrovan V., Sheahan M., Cino-Ozuna A.G., Fang Y., Hesse R., Mileham A., Samuel M.S., Wells K.D. (2019). Resistance to coronavirus infection in amino peptidase N-deficient pigs. Transgenic. Res..

[36] Annamalai T., Saif L.J., Lu Z., Jung K. (2015). Age-dependent variation in innate immune responses to porcine epidemic diarrhea virus infection in suckling versus weaned pigs. Vet. Immunol. Immunopathol..

[37] Jung K., Miyazaki A., Saif L.J. (2018). Immunohistochemical detection of the vomiting-inducing monoamine neurotransmitter serotonin and enterochromaffin cells in the intestines of conventional or gnotobiotic (Gn) pigs infected with porcine epidemic diarrhea virus (PEDV) and serum cytokine responses of Gn pigs to acute PEDV infection. Res. Vet. Sci..

[38] Gao Q., Zhao S., Qin T., Yin Y., Yang Q. (2015). Effects of porcine epidemic diarrhea virus on porcine monocyte-derived dendritic cells and intestinal dendritic cells. Vet. Microbiol..

[39] Jung K., Saif L.J., Wang Q. (2020). Porcine epidemic diarrhea virus (PEDV): An update on etiology, transmission, pathogenesis, and prevention and control. Virus Res..

[40] Yuan C., Sun L., Chen L., Guo H., Yao Z., Wang Y., Zhu W., Li T., Song Q., Li H. (2022). Chemokines induced by PEDV infection and chemotactic effects on monocyte, T and B cells. Vet. Microbiol..

[41] Hu Z., Li Y., Du H., Ren J., Zheng X., Wei K., Liu J. (2020). Transcriptome analysis reveals modulation of the STAT family in PEDV-infected IPEC-J2 cells. BMC Genomics.

